# Efficacy and safety of cryoballoon pulmonary vein isolation for paroxysmal and persistent atrial fibrillation: A comparison with radiofrequency ablation

**DOI:** 10.1371/journal.pone.0265482

**Published:** 2022-07-27

**Authors:** Ji-Hoon Choi, Seung-Jung Park, Kyoung-Min Park, June Soo Kim, Young Keun On

**Affiliations:** Division of Cardiology, Heart Vascular and Stroke Institute, Samsung Medical Center, Sungkyunkwan University School of Medicine, Seoul, Republic of Korea; University of Minnesota, UNITED STATES

## Abstract

**Background:**

Cryoballoon ablation was established as an effective and safe modality to achieve pulmonary vein isolation (PVI) in paroxysmal atrial fibrillation (PAF). However, its role in persistent atrial fibrillation (PersAF) remains unclear.

**Objective:**

This study aimed to evaluate the efficacy and safety of cryoballoon PVI in PAF and PersAF comparing conventional radiofrequency catheter ablation (RFCA).

**Methods:**

Two hundred patients undergoing cryoballoon ablation for symptomatic AF were consecutively enrolled in this retrospective study. For comparison, 210 patients undergoing RFCA in the same period were included. The primary outcome was a recurrence of any atrial tachyarrhythmias (ATas) after the index ablation. 12-lead ECG and 24-hour Holter monitoring were obtained at 1,3,6 and 9–12 months.

**Results:**

PVI by cryoablation alone was achieved in 197 patients (98.5%). ATas-free survival at 12 months post-ablation was 72.7% in the cryoablation and 80.6% in the RFCA group (P = 0.123), respectively. The cryoablation showed comparable efficacy maintaining sinus rhythm compared with RFCA in PAF (P = 0.539), whereas in PersAF, ATas-free survival was significantly lower in cryoablation (P = 0.039). PV reconnection was observed in the majority of patients (14/16, 87.5%) who receive redo RFCA. Complications were encountered in 10 patients, including femoral arteriovenous fistula (n = 1), transient phrenic nerve palsy (n = 8), and minimal amount pericardial effusion (n = 1).

**Conclusion:**

The efficacy of cryoballoon PVI is comparable with conventional RFCA in PAF, whereas PVI alone using cryoballoon may not be insufficient to maintaining sinus rhythm in PersAF. The safety of cryoballlon PVI is tolerable.

## Introduction

Atrial fibrillation (AF) is a chronic disorder that begins as paroxysmal and progresses to persistent and perpetual [1]. Five to 10% of paroxysmal AF (PAF) patients progress to persistent AF (PersAF) per year [2]. AF itself causes atrial electrical remodeling, which deteriorates the structural remodeling process and ultimately creates the AF substrate [3]. In other words, AF makes it more challenging to maintain the sinus rhythm over time. For this reason, the early rhythm control strategy has been widely accepted in clinical practice, with evidence supporting its ability to reduce cardiovascular events [4]. Antiarrhythmic drugs (AADs) remain the first-line rhythm control therapy for symptomatic AF [5]. However, randomized controlled trials have demonstrated the superiority of catheter ablation over AAD in maintaining sinus rhythm [6, 7].

Electrical pulmonary vein isolation (PVI) is the cornerstone of catheter-based ablation for AF [5]. In particular, cryoballoon ablation was established as a safe and effective modality for achieving PVI in PAF compared with radiofrequency catheter ablation (RFCA) [8, 9]. The simplicity of the single-shot technique and relatively short learning curve make cryoballoon ablation a promising procedure that has gained prevalence in clinical practice for PAF. However, its role in PersAF remains unclear, and an optimal ablation strategy has yet to be determined. The aim of this study is to evaluate the efficacy and safety of cryoballoon PVI for PAF and PersAF in comparison to conventional RFCA guided by a 3D navigation system.

## Method

### Study population

This study complied with the Declaration of Helsinki, and the research protocol was approved by Samsung Medical Center Institutional Review Board (SMC IRB File No. 2021-04-148-001). Before initiating this retrospective study, the SMC IRB waived the informed consent requirement. During the conduct of this study, all medical records of patients were fully anonymized. We conducted this retrospective study from April 2021 to May 2021.

Since June 2014, clinical, electrocardiographic, echocardiographic, and procedure-related variables from consecutive patients undergoing cryoablation and RFCA at our institution have been prospectively collected and entered into a procedural database and electronic medical record. All patients ≥18 years old undergoing cryoballoon ablation for symptomatic AF from June 2018 to May 2020 were consecutively included in this retrospective study. For comparison with cryoablation, all consecutive patients undergoing RFCA for symptomatic AF from June 2018 to December 2019 were screened for eligibility. Patients with a history of previous left atrial (LA) ablation or cardiac surgery were excluded. Indications for catheter ablation include at least 2 episodes (each lasting >30 s) of PAF or 1 episode of PersAF. Before ablation, failure with at least one class I or class III AAD was documented. PAF was defined as AF lasting less than 7 days with spontaneous termination, and PersAF was defined as AF lasting longer than 7 days without spontaneous termination or AF requiring cardioversion.

### Pre-procedural evaluation

All patients were treated with a non-vitamin K antagonist oral anticoagulants (NOACs) for at least 3 weeks before the procedure. To rule out intra-atrial thrombus, we routinely performed transesophageal echocardiography (together with transthoracic echocardiography) less than 24 hours before the procedure. The left atrial (LA) diameter was measured in the M-mode parasternal long-axis view at the end of the left ventricle (LV) systole [10]. The LA volume index (LAVI) was measured by the biplane area length method using the apical four-chamber and apical two-chamber view at the end-systole and indexed to the calculated body surface area using the Du Bois formula [10]. The LV ejection fraction (LVEF) was accessed by the biplane Simpson method at ventricular end-diastole [10]. All patients underwent cardiac computed tomography (CT) with 3-dimensional (3D) surface reconstruction of the LA, LA appendage, and pulmonary veins one day before the procedure. Ablation modality was determined without information on LA and pulmonary vein (PV) anatomy on an outpatient basis and was not altered by cardiac CT information afterward.

### Cryoballoon ablation procedure

All procedures were conducted under general anesthesia with esophageal temperature monitoring under the supervision of an anesthesiologist. Two punctures for each femoral vein were performed for cryoballoon ablation. Diagnostic electrophysiology catheters, a duodecapolar (located at the right atrium and coronary sinus) and a deflectable quadripolar catheter (located at the right ventricle) were placed through a left femoral vein. Intracardiac echocardiography (ICE) was routinely used to guide a single transseptal puncture. After obtaining LA access, unfractionated heparin was administered at 100 IU/kg. The activated clotting time was checked every 30 minutes for a target level of more than 300 s. A steerable 15-Fr sheath (FlexCath Advance^TM^, Medtronic, Minneapolis, MN) was introduced into the LA. Then, a 28-mm second-generation cryoballoon (Arctic Front Advance^TM^, Medtronic) was advanced toward each PV ostium by moving the balloon catheter over an inner lumen circular mapping catheter (Achieve^TM^, Medtronic). PV potentials (PVPs) were recorded using a mapping catheter at a PV ostium before ablation. After balloon inflation, operators re-positioned the catheter to seal the PV ostium completely under fluoroscopic guidance. Complete PV occlusion was documented using selective contrast injection before ablation. Freeze duration depended on the time to PV isolation (TTI): 1) if TTI was shorter than 60 s, cryoapplication lasted for 180 s, 2) if TTI was longer than 60 s or PVPs were not measured, a bonus freeze of 120 s was applied at the operator’s discretion. If AF persisted after complete PVI, the sinus rhythm was restored by internal electrical cardioversion. After PV cryoablation, bi-directional PVI was confirmed in the sinus rhythm at a proximal site in the PV ostium. When PVI was not achieved by cryoablation, touch-up RFCA was performed. Right phrenic nerve pacing (10–20 mA, 800–1200 ms) using a deflectable quadripolar catheter was performed to recognize phrenic nerve palsy during right PV ablation. If cavotricuspid isthmus (CTI)-dependent atrial flutter had previously been documented, concomitant CTI ablation using a radiofrequency catheter was allowed.

### Radiofrequency catheter ablation procedure

Preparation for RFCA followed the same procedure as for cryoballoon ablation except for one more femoral puncture to perform double transseptal puncture. After the 3D mapping of the LA geometry with ICE (SOUNDSTAR^®^, Biosence Webster, Diamond Bar, CA), the map was integrated with the previously acquired cardiac CT. A variable circular mapping catheter (LASSO^TM^, Biosence Webster) was introduced into the LA, and electroanatomic mapping of the LA was performed with the guidance of a 3D navigation system (CARTO^®^, Biosence Webster). A quadripolar deflectable 3.5-mm irrigated-tip catheter with contact-force monitoring (THERMOCOOL SMARTTOUCH^®^ SF catheter, Biosense Webster) was advanced into each PV antrum. PV isolation was achieved by creating a wide antral circumferential ablation lesion under CARTO guidance. PVI alone was performed in PAF patients, but posterior wall isolation (PWI) in conjunction with PVI was performed selectively in PersAF patients at the operator’s discretion.

### Study outcome and follow-up

The primary outcome of this study was the recurrence of any atrial tachyarrhythmia (AF, atrial flutter, or atrial tachycardia) after the ablation during the follow-up period. A diagnosis of atrial tachyarrhythmia (ATa) recurrence was made when ATas were confirmed on 12-lead ECG or ATas lasting at least 30 s were documented on Holter monitoring. Early recurrence was defined as recurrence within the first 3 months post-ablation, known as the blanking period, and was recorded but not considered to indicate a primary outcome failure.

After the index ablation procedure, clinical follow-up was scheduled at 1, 3, 6, and 12 months. At each visit, patients were asked about the recurrence of AF-related symptoms, and a 12-lead ECG was obtained. 24-hour Holter monitoring was applied at 3, 6, and 9–12 months. Patients who had recurrent AF-related symptoms were evaluated immediately using Holter or event monitoring, regardless of the follow-up schedule, and they were told to receive a 12-lead ECG at a nearby hospital as soon as symptoms occurred. NOAC and an AAD (class I or class III) were continued for at least 3 months and individualized after that at the clinician’s discretion.

### Statistical analysis

Continuous variables are reported as mean value ± standard deviation (SD) or median with interquartile ranges, and all categorical variables are given as the number and percentage. We used the Student’s t-test or Mann-Whitney test to compare continuous variables between the two groups and the chi-square or Fisher’s exact test to compare categorical variables. The ATas-free survival analysis was performed using the Kaplan-Meier method. The log-rank test was performed to compare the survival curves between the groups. Univariate and multivariate Cox regression analyses were conducted to identify the predictors of ATa recurrence after cryoablation. All statistical tests were two-tailed, and a P-value of <0.05 was considered significant. All statistical analyses were performed using SPSS statistical software version 25 (SPSS Inc., Chicago, IL).

## Results

### Baseline characteristics

**Fig 1:**
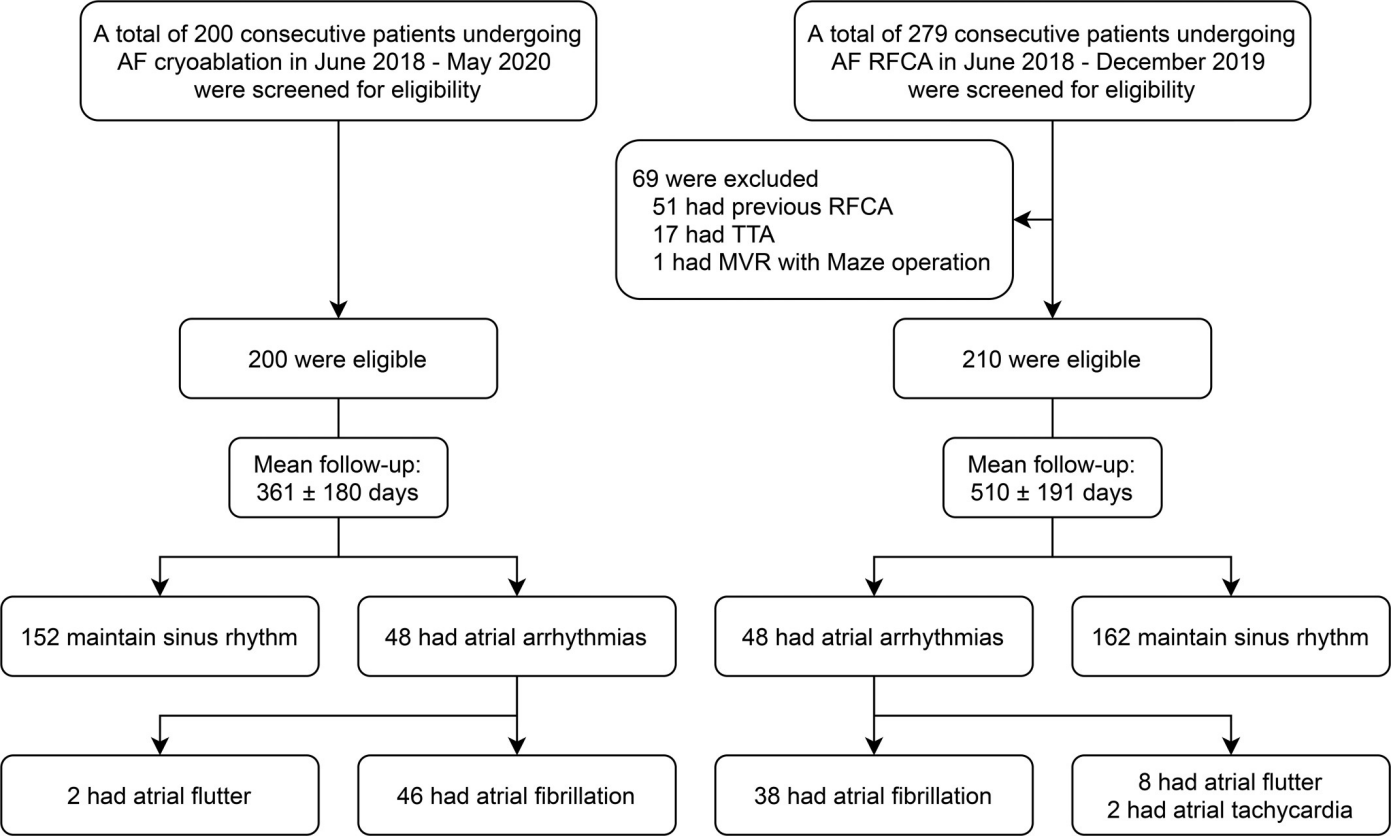
Study flow diagram with the number of patients.

The second-generation cryoballoon was first used at our institution in June 2018. In the next two years, 200 consecutive patients underwent cryoablation for symptomatic AF. Meanwhile, RFCA was performed in 279 patients with symptomatic AF in a similar period. Among the RFCA patients, 69 were excluded from this study based on our exclusion criteria (Fig 1). Thus, 210 patients undergoing RFCA were included in this study. The baseline clinical characteristics of the study population are summarized in Table 1 according to ablation modality. The mean age was similar in the cryoablation *vs*. RFCA groups (57.5 ± 9.8 years *vs*. 56.6 ± 10; P = 0.366), and male sex was dominant in both groups (P = 0.140). The proportion of PersAF patients did not differ between the two groups (Cryoablation *vs*. RFCA; 64 [32.0%] *vs*. 79 [37.6%]; P = 0.233). More patients in the cryoablation group (6.0%) than in the RFCA group (1.9%) had a history of congestive heart failure (P = 0.032). The median time from the first documented AF episode to ablation was significantly longer in the cryoablation group (2.3 years [1.2–4.5]) than in the RFCA group (1.4 years [0.9–2.8]) (P <0.001). The two groups did not differ significantly in other variables: comorbidities, LV systolic function, LA size, and medication use (except for beta-blockers, Cryoablation *vs*. RFCA; 21 [10.5%] *vs*. 40 [19.0%]; P = 0.015).

**Table 1:** Baseline characteristics by ablation modality.

Variables	Cryoablation group (n = 200)	RFCA group (n = 210)	P-value
Age, years	57.5 ± 9.8	56.6 ± 10	0.366
Age ≥65 years, n (%)	50 (25.0)	44 (21.0)	0.330
Sex, male, n (%)	153 (76.5)	173 (82.4)	0.140
BMI, kg/m^2^	25.5 ± 2.9	25.3 ± 2.8	0.511
Diabetes mellitus, n (%)	25 (12.5)	29 (13.8)	0.695
Hypertension, n (%)	88 (44.0)	105 (50.0)	0.224
Prior stroke/TIA, n (%)	13 (6.5)	15 (7.1)	0.796
Prior congestive heart failure, n (%)	12 (6.0)	4 (1.9)	**0.032**
Prior myocardial infarction, n (%)	2 (1.0)	3 (1.4)	1.000
Persistent AF, n (%)	64 (32.0)	79 (37.6)	0.233
Longstanding persistent AF, n (%)	16 (8.0)	19 (9.0)	0.704
First AF episode to ablation, years	2.3 (1.2–4.5)	1.4 (0.9–2.8)	**<0.001**
First AF episode to ablation ≥1 year	158 (79.0)	154 (73.3)	0.179
CHA_2_DS_2_VASc score			
Mean	1.3 ± 1.2	1.2 ± 1.1	0.717
Distribution			0.712
0	63 (31.5)	64 (30.5)	
1	63 (31.5)	76 (36.2)	
2	43 (21.5)	37 (17.6)	
3	23 (11.5)	28 (13.3)	
4	6 (3.0)	3 (1.4)	
5	2 (1.0)	2 (1.0)	
LVEF, %	61.1 ± 7.1	61.8 ± 6.1	0.311
LA diameter, mm	42.3 ± 6.2	42.2 ± 6.0	0.834
LA diameter ≥40 mm, n (%)	131 (65.5)	133 (63.3)	0.647
LA volume index, ml/m^2^	41.2 ± 12.5	41.3 ± 13.7	0.991
LA volume index ≥35 ml/m^2^	136 (68.0)	127 (60.5)	0.112
Medication			
AAD during blanking period, n (%)	163 (81.5)	162 (77.1)	0.277
AAD after blanking period, n (%)	55 (27.5)	58 (27.6)	0.978
Beta-blocker, n (%)	21 (10.5)	40 (19.0)	**0.015**
ACE inhibitor or ARB, n (%)	45 (22.5)	53 (25.2)	0.516
Anticoagulation drug, n (%)	164 (82.0)	157 (74.8)	0.076

Values are expressed as n (%), mean ± SD, or median with interquartile range.

RFCA = radiofrequency catheter ablation; BMI = body mass index; TIA = transient ischemic attack; AF = atrial fibrillation; LVEF = left ventricular ejection fraction; LA = left atrium; ACE = angiotensin-converting enzyme; ARB = angiotensin-receptor blocker; CHA_2_DS_2_VASc score = congestive heart failure, hypertension, age ≥75 years, diabetes mellitus, prior stroke or transient ischemic attack or thromboembolism, vascular disease, age 65–74 years, sex category; AAD = antiarrhythmic drug.

### Procedural characteristics of cryoablation

PVI was achieved by cryoablation alone in 197 patients (98.5%). In the early learning period, touch-up RFCA was performed in 2 patients because PVI was not achieved by cryoablation and in 1 patient because the right inferior PV (RIPV) was inaccessible. Additional RFCA confirmed complete PVI in those patients. The mean freeze time per vein was 293.1 ± 93.8 s. PVI during cryoablation was observed less in the RIPV than in the other PVs: left superior PV (LSPV), 73.8%; left inferior PV (LIPV), 53.0%; right superior PV (RSPV), 56.6%; RIPV, 27.3%. The mean procedure and fluoroscopy times were 82.9 ± 20.9 and 26.7 ± 10.5 min, respectively. The mean LA dwelling time, meaning the time from the septal puncture to the end of the ablation, was 53.5 ± 10.9 min.

Complications were encountered in 10 patients (5.0%). Only 1 major complication required endovascular stent-graft repair due to a femoral arteriovenous fistula at a vascular access site. Transient phrenic nerve palsy occurred in 8 patients (4.0%) but recovered completely by the end of the procedure. A minimal amount of pericardial effusion was observed in 1 patient, and it disappeared spontaneously at follow-up echocardiography. Detailed procedural characteristics for the cryoablation are summarized in Table 2.

**Table 2:** Procedural characteristics of cryoablation.

Cryoballoon applications (per patient)	7.5 ± 2.5
LSPV	2.0 ± 0.9
LIPV	1.9 ± 1.3
RSPV	1.8 ± 1.1
RIPV	1.8 ± 1.1
Mean application time (per vein)	293.1 ± 93.8
Nadir balloon temperature, °C	
LSPV	- 50.0 ± 5.9
LIPV	- 44.5 ± 5.3
RSPV	- 51.3 ± 6.2
RIPV	- 46.3 ± 7.7
PV isolation during procedure, n (%)	
LSPV	155 (78.3)
LIPV	105 (53.0)
RSPV	112 (56.6)
RIPV	54 (27.3)
Time to isolation when observed, s	
LSPV	57.7 ± 31.6
LIPV	47.0 ± 32.7
RSPV	36.2 ± 30.6
RIPV	49.0 ± 39.9
Procedural time, min	82.9 ± 20.9
Fluoroscopy time, min	26.7 ± 10.5
LA dwelling time, min	53.5 ± 16.9
Complication, n (%)	10 (5.0)
Phrenic nerve palsy, n (%)	8 (4.0)
Transient, n (%)	8 (4.0)
Persistent, n (%)	0
Arteriovenous fistula, n (%)	1 (0.5)
Pericardial effusion, n (%)	1 (0.5)
3-dimensional mapping, n (%)	3 (1.5)

LSPV = left superior pulmonary vein; LIPV = left inferior pulmonary vein; RSPV = right superior pulmonary vein; RIPV = right inferior pulmonary vein; LA = left atrium.

### Primary outcome after the index procedure

**Fig 2:**
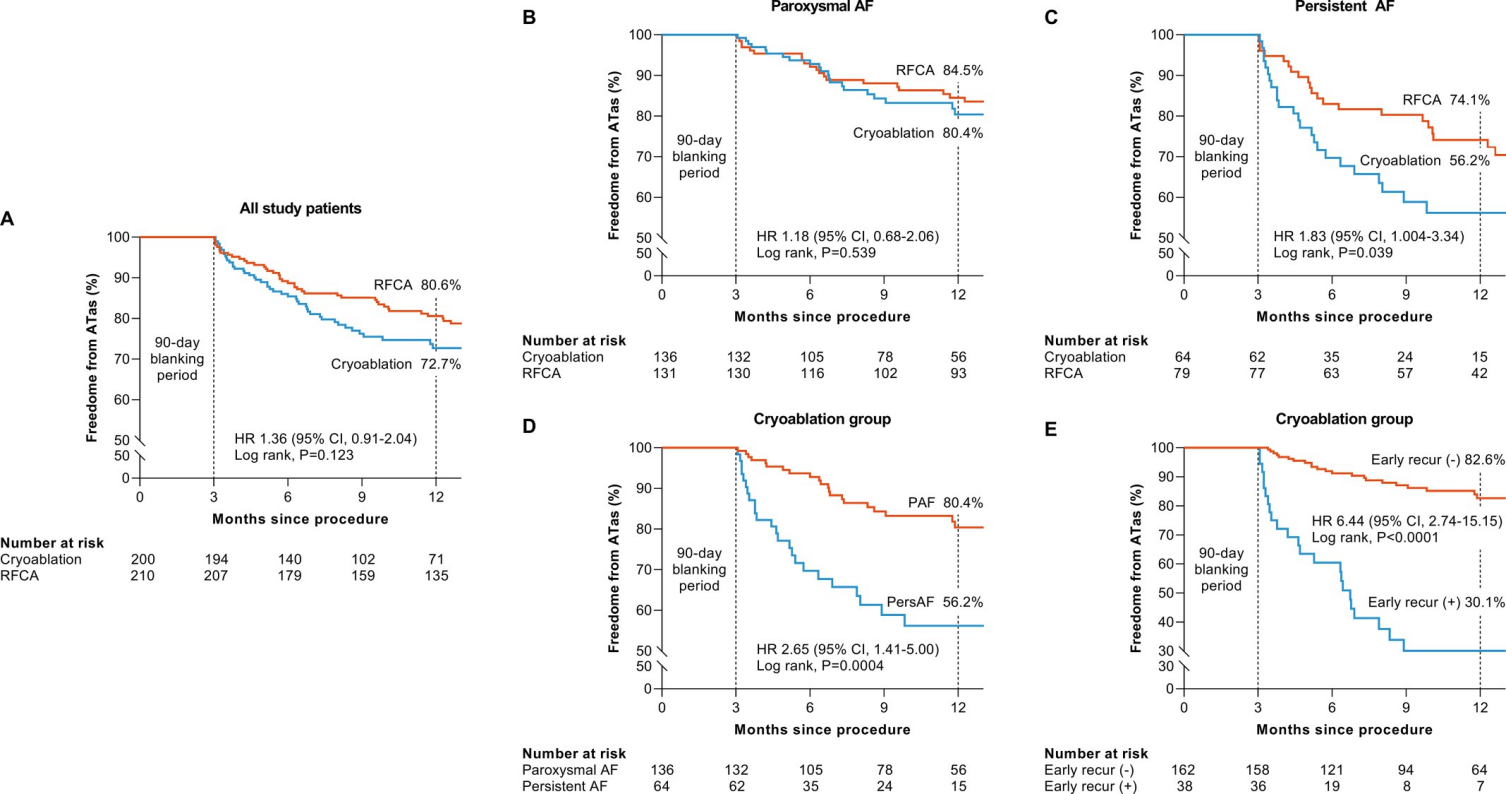
Kaplan-Meier estimates of atrial tachyarrhythmia (ATa) free survival. **(A)** freedom from ATa in all study population according to ablation modality, **(B)** freedom from ATa in paroxysmal AF according to ablation modality, **(C)** freedom from ATas in persistent AF according to ablation modality, **(D)** freedom from ATa in cryoablation group according to AF type, **(E)** freedom from ATa in cryoablation group according to early recurrence. AF = atrial fibrillation.

The mean follow-up duration was significantly longer in the RFCA group (510 ± 191 days) than in the cryoablation group (361 ± 180 days) (P = 0.002) because more patients who received the procedure relatively late in the study period were included in the cryoablation group. At 12 months post-ablation, the Kaplan-Meier estimate of freedom from ATas in all study patients was 72.7% for cryoablation and 80.6% for RFCA. Forty-eight patients in each ablation group had ATa recurrence during the follow-up period. Cryoablation showed ATas-free survival comparable to that with RFCA (HR: 1.36; 95% CI: 0.91–2.04; P = 0.123) in all AF patients (Fig 2A). However, ATas-free survival did differ between the two groups in the subgroup analysis stratified by AF type. In PAF patients, freedom from ATas after cryoablation did not differ significantly from that after RFCA (HR: 1.18; 95% CI: 0.68–2.06; P = 0.539) (Fig 2B). In PersAF patients, on the other hand, freedom from ATas in the cryoablation group was significantly lower than in the RFCA group at the 12-month follow-up (HR: 1.83; 95% CI: 1.004–3.34; P = 0.039) (Fig 2C). ATa recurrence was more frequently observed in PersAF patients than in PAF patients regardless of ablation modality, but a significant difference was observed only in the cryoablation group at 12 months (PAF vs. PersAF; 80.4% vs. 56.2%; HR: 2.65; 91% CI: 1.41–5.00; P = 0.0004) (Fig 2D). ATas-free survival was significantly lower in patients with early recurrence than in patients without early recurrence (HR: 6.44; 95% CI: 2.74–15.15; P < 0.0001) (Fig 2E).

The type of ATa recurrence also differed significantly between the cryoablation and RFCA groups (Table 3). AF was the most common type of ATa recurrence in both groups. However, the other ATas, atrial flutter and atrial tachycardia, occurred more often in the RFCA group (10/48, 20.8%) than in the cryoablation group (2/48, 4.2%). After a subgroup analysis stratified by AF type, that difference was observed only in PersAF patients.

**Table 3:** Type of atrial arrhythmia recurrence after index procedure.

**Atrial arrhythmias**	**Cryoablation group (n = 48)**		**RFCA group (n = 48)**		**P-value**	
Atrial fibrillation	46 (95.8)		38 (79.2)		0.014	
Other	2 (4.2)		10 (20.8)			
Atrial flutter	2 (4.2)		8 (16.7)			
Atrial tachycardia	0		2 (4.2)			
**Atrial arrhythmias**	**Paroxysmal AF**			**Persistent AF**		
	**CRYO (n = 24)**	**RFCA (n = 27)**	**P-value**	**CRYO (n = 24)**	**RFCA (n = 21)**	**P-value**
Atrial fibrillation, n (%)	22 (91.7)	21 (77.8)	0.255	24 (100)	17 (81.0)	0.040
Other, n (%)	2 (8.3)	6 (22.2)		0 (0)	4 (19.0)	
Atrial flutter	2	6		0	2	
Atrial tachycardia	0	0		0	2	

AF = atrial fibrillation; CRYO = cryoablation; RFCA = radiofrequency catheter ablation.

### Results of redo ablation

Among patients with ATa recurrence after AF ablation, 16 out of 48 patients (33.3%) in the cryoablation group and 21 out of 48 patients (43.8%) in the RFCA group underwent redo ablation using a radiofrequency catheter due to refractory AF-related symptoms despite continuing AAD. The redo ablation rate was similar between the two groups (Cryoablation vs. RFCA; 16/200 [8.0%] vs. 21/210 [10.0%]; P = 0.480). Among the patients who underwent redo ablation, the PV reconnection rate tended to be higher in the cryoablation group (Cryoablation vs. RFCA; 14/16 [87.5%] vs. 13/21 [61.9%]; P = 0.137).

Both carinas and the left ridge were common PV reconnection sites in both groups. However, in the cryoablation group, PV reconnection was frequently observed in the RIPV inferior (50.0%) but not in the RFCA group (9.5%). Conversely, PV reconnection in the roof of superior PVs was relatively common in the RFCA group (33.3%) but not in the cryoablation group (6.3%). The specific location of LA-PV reconnection in patients who underwent redo RFCA is presented in Table 4.

**Table 4:** The specific location of LA-PV reconnection in patients who underwent redo RFCA.

Location of PV gap	Cryoablation (n = 16)	RFCA (n = 21)
RIPV, n (%)	14 (87.5)	6 (28.6)
Inferior, n (%)	8 (50.0)	2 (9.5)
Carina, n (%)	6 (37.5)	4 (19.0)
LSPV, n (%)	8 (50.0)	8 (38.1)
Carina, n (%)	4 (25.0)	2 (9.5)
Ridge, n (%)	3 (18.0)	3 (14.3)
Superior, n (%)	1 (6.3)	3 (14.3)
LIPV, n (%)	6 (37.5)	4 (19.0)
Ridge, n (%)	3 (18.8)	1 (4.8)
Inferior, n (%)	3 (18.8)	0
Carina, n (%)	0	3 (14.3)
RSPV, n (%)	4 (25.0)	7 (33.3)
Carina, n (%)	4 (25.0)	3 (14.3)
Superior, n (%)	0	4 (19.0)

LA = left atrium; RFCA = radiofrequency catheter ablation; PV = pulmonary vein; RIPV = right inferior pulmonary vein; RSPV = right superior pulmonary vein; LSPV = left superior pulmonary vein; LIPV = left inferior pulmonary vein.

### Predictors of ATa recurrence after cryoablation

The baseline clinical and procedural characteristics of the cryoablation group are summarized in S1 File according to ATa recurrence. ATa recurrence after cryoablation was significantly associated with PersAF, LA diameter ≥40 mm, and early ATa recurrence (Table 5). Age and variables with a P-value <0.1 after the univariate Cox regression analysis were selected for the multivariate analysis. In that analysis, PersAF (HR: 1.833; 95% CI: 1.014–3.313; P = 0.045), LA diameter ≥40 mm (HR: 3.305; 95% CI: 1.448–7.542; P = 0.005), and early ATa recurrence (HR: 5.010; 95% CI: 2.713–9.254; P <0.001) were independent predictors of ATa recurrence after cryoablation (Table 5).

**Table 5:** Univariate and multivariate Cox regression analyses for predictors of atrial tachyarrhythmia recurrence after cryoablation.

	Univariate		Multivariate	
Variables	HR (95% CI)	P-value	HR (95% CI)	P-value
Age	0.994 (0.965–1.024)	0.686		
Age ≥65 years	0.670 (0.324–1.383)	0.279	0.521 (0.248–1.092)	0.084
Male	1.303 (0.648–2.619)	0.458		
BMI	1.067 (0.979–1.162)	0.142		
BMI ≥30 kg/m^2^	2.010 (0.851–4.752)	0.112		
Hypertension	1.051 (0.596–1.855)	0.863		
Diabetes	1.142 (0.485–2.689)	0.762		
Prior stroke/TIA	0.589 (0.143–2.428)	0.464		
Prior CHF	0.629 (0.153–2.590)	0.520		
Persistent AF	2.667 (1.512–4.704)	0.001	1.833 (1.014–3.313)	**0.045**
AF duration	1.024 (0.928–1.130)	0.630		
AF duration ≥1 year	1.516 (0.680–3.379)	0.309		
CHA_2_DS_2_VASc score	0.904 (0.703–1.162)	0.431		
LA diameter ≥40 mm	3.776 (1.692–8.426)	0.001	3.305 (1.448–7.542)	**0.005**
LAVI ≥35 ml/m^2^	2.034 (1.013–4.084)	0.046		
Early recurrence	6.848 (3.821–12.273)	<0.001	5.010 (2.713–9.254)	**<0.001**

BMI = body mass index; TIA = transient ischemic attack; CHF = congestive heart failure; AF = atrial fibrillation; CHA_2_DS_2_VASc score = congestive heart failure, hypertension, age ≥75 years, diabetes mellitus, prior stroke or transient ischemic attack or thromboembolism, vascular disease, age 65–74 years, sex category; LA = left atrium; LAVI = left atrial volume index.

## Discussion

### Main findings

We compared the efficacy and safety of cryoballoon PVI with RFCA in PAF and PersAF patients. The main findings of this study are: (1) cryoballoon PVI showed efficacy with regard to ATa recurrence comparable to that with RFCA in PAF patients, whereas in PersAF, cryoballoon PVI was found to be inferior to RFCA; (2) the type of ATa recurrence differed by ablation modality; (3) PV reconnection can be a major cause of AF recurrence after cryoablation, and the RIPV inferior, carina, and left ridge were common sites of PV reconnection; (4) early ATa recurrence during the 3-month blanking period was the most powerful predictor of late ATa recurrence after cryoablation.

### PV reconnection as a major cause of AF recurrence after cryoablation

A larger isolated antral surface area has been shown to be closely associated with a significantly lower AF recurrence rate [11]. Meanwhile, Kenigsberg et al. reported that PVI using the 28-mm second-generation cryoballoon creates a wide antral lesion set with posterior LA wall debulking [12]. In our study, AF-free survival for PersAF patients was significantly lower in the cryoablation group than in the RFCA group (HR: 2.21; 95% CI: 1.18–4.14; P = 0.0096) (S1 Fig). What made that difference in ATas recurrence if the ablation modalities did not differ in the isolated antral area?

Atrial tissue fibrosis is a key structural change that makes cardiomyocyte arrhythmogenic contributing to therapeutic resistance in atrial fibrillation [3]. The increased burden of atrial fibrosis detecting by delayed enhancement magnetic resonance imaging has been shown to be higher in PersAF compared with PAF and to be associated with more ATas recurrence [13, 14]. Furthermore, the burden of atrial fibrosis was closely related to the extent of PV reconnection [15]. In our study, more cryoballoon applications (8.0 ± 2.6 vs. 7.2 ± 2.4; P = 0.035) and longer freezing times (1286 ± 450 vs. 1119 ± 323 sec; P = 0.004) were required to achieve PVI in PersAF compared to PAF. That may implicate that a higher fibrosis burden in PersAF hinders cryothermal energy delivery resulting in PV reconnection. Our result showed that PV reconnection was commonly observed (14/16, 87.5%) in patients who received redo RFCA. Several studies have shown that AF recurrence after cryoablation was mainly but not entirely caused by PV reconnection [16, 17]. Moreover, Wieczorek et al. reported that more extensive PV reconnections result in earlier AF recurrence after cryoablation [17]. In our study, PV reconnection was frequently observed at the right carina (62.5%) and the left ridge and carina (62.5%). Similarly, previous studies revealed that the carina and left ridge regions are the most common sites of PV reconnection [16, 17]. Relatively thick tissue in those areas makes it difficult to attain durable PVI especially in PersAF. Those findings suggest that AF recurrence after cryoablation might not be due to a lack of substrate modification in the antrum but instead be caused by incomplete transmural lesion. Furthermore, we made the interesting finding that the type of recurrent ATa differed according to the ablation modality. In the cryoablation group, ATa recurrence presented as AF in all but 2 patients (95.8%). In the RFCA group, on the other hand, atypical atrial flutter and atrial tachycardia accounted for 20.9% of ATa recurrence. This finding also reflects that PV reconnection is a major reason for the low AF-free survival rate after cryoablation. Bonus freezing of the carina and ridge region could help to achieve durable PVI especially in PersAF.

Previous studies reported that the RIPV is one of the preferential PV reconnection sites after cryoablation [18, 19]. In our study, the RIPV was also the most common site of PV reconnection. The closeness of the RIPV to the transeptal puncture site makes it difficult to completely attach the cryoballoon to the PV ostium, especially in RIPVs with more inferior angles. Knowledge of precise PV anatomy through preprocedural cardiac CT and an integrated approach could help to achieve durable PVI in the RIPV [20].

Additionally, the time from initial diagnosis of AF to ablation is closely associated with AF recurrence post-ablation [21]. In patients with PersAF, a relatively long time from diagnosis to ablation (Cryoablation vs. RFCA; 2.1 years [1.2–4.8] vs. 1.4 years [1.1–2.6]; P = 0.043) may have affected the worse outcome.

### Predictors for atrial tachyarrhythmia recurrence after cryoablation

According to the current expert consensus statement, early recurrence (within 3 months post-ablation, known as the blanking period) should not be classified as treatment failure [22]. That is because the acute inflammatory process caused by ablation can induce AF recurrence in that early period [23]. However, Stabile et al. suggested that AF recurrence in the blanking period is a strong predictor of AF recurrence. Similarly, early ATa recurrence was the most potent predictor of late ATa recurrence in this study after adjusting for age, sex, AF type, and LA diameter. Patients with early recurrence were 5 times more likely to develop ATas than those without early recurrence. More attention and a shorter follow-up schedule for ECG monitoring should be considered in patients with early recurrence.

### Limitations

This study was a non-randomized, single-center study with a relatively short follow-up time. Furthermore, the number of patients with PersAF was small. A large RCT with a long follow-up duration is warranted. ATa recurrence might have been underestimated because we counted it only when it was diagnosed by 24-hour Holter monitoring, an event recorder, or 12-lead ECG; we did not use long-term continuous monitoring, such as with an implantable loop recorder. Because the ablation strategies in PersAF differed between the ablation modalities, direct comparison of primary outcomes should be made with caution.

## Conclusions

The efficacy of cryoballoon PVI is comparable to PVI using RFCA in PAF, whereas PVI alone using a cryoballoon might not be sufficient to maintain the sinus rhythm in PersAF. PV reconnection is a presumably dominant cause of AF recurrence after cryoablation, and the RIPV inferior, both carinas, and left ridge were common sites of PV reconnection. More attention should be paid to patients with early recurrence due to their high risk for late recurrence.

## Supporting information

S1 Checklist STROBE Statement—checklist of items that should be included in reports of observational studies. (DOCX)

S1 File Baseline and procedural characteristics according to atrial tachyarrhythmia recurrence in the cryoablation group. (DOCX)

S1 Fig Kaplan-Meier estimates of atrial fibrillation free survival. (TIF)

S1 Raw data (XLSX)
