# Supplementary material for: Efficacy and safety of cryoballoon pulmonary vein isolation for paroxysmal and persistent atrial fibrillation: A comparison with radiofrequency ablation
Source: PLoS One. 2022 Jul 27;17(7):e0265482. doi: 10.1371/journal.pone.0265482 (PMC9328506; doi:10.1371/journal.pone.0265482)
Supplement: S1 File — (DOCX) [file pone.0265482.s002.docx]

**S1 Table.** Baseline and procedural characteristics according to atrial tachyarrhythmia recurrence in the cryoablation group

| **Variables** | **No recurrence**  **(N=152)** | **Recurrence**  **(N=48)** | **P-value** |
| --- | --- | --- | --- |
| Follow-up duration, days | 344.5 ± 175.9 | 411.1 ± 186.6 | **0.025** |
| Age, years | 57.6 ± 10.2 | 57.3 ± 8.6 | 0.867 |
| Age ≥65 years, n (%) | 41 (27.0) | 9 (18.8) | 0.251 |
| Sex, male, n (%) | 115 (75.7) | 38 (79.2) | 0.617 |
| BMI, kg/m^2^ | 25.3 ± 2.7 | 26.1 ± 3.4 | 0.104 |
| Hypertension, n (%) | 65 (42.8) | 23 (47.9) | 0.531 |
| Diabetes mellitus, n (%) | 19 (12.5) | 6 (12.5) | 1.000 |
| Prior stroke/TIA, n (%) | 11 (7.2) | 2 (4.2) | 0.737 |
| Prior congestive heart failure | 10 (6.6) | 2 (4.2) | 0.734 |
| Prior myocardial infarction | 2 (1.3) | 0 | 1.000 |
| Persistent AF, n (%) | 40 (26.3) | 24 (50.0) | **0.002** |
| First AF episode to ablation, years | 2.3 (1.1 – 4.5) | 2.4 (1.5 – 4.7) | 0.462 |
| First AF episode to ablation ≥1 year | 117 (77.0) | 41 (85.4) | 0.211 |
| CHA_2_DS_2_VASc score | 1.3 ± 1.2 | 1.2 ± 1.0 | 0.526 |
| CHA_2_DS_2_VASc score ≥2, n (%) | 57 (37.5) | 17 (35.4) | 0.794 |
| LVEF, % | 61.3 ± 7.3 | 60.5 ± 6.3 | 0.496 |
| LA diameter, mm | 41.7 ± 6.4 | 44.2 ± 5.2 | **0.008** |
| LA diameter ≥40 mm, n (%) | 90 (59.2) | 41 (85.4) | **0.001** |
| LA volume index, ml/m^2^ | 40.5 ± 12.8 | 43.7 ± 11.5 | 0.116 |
| LA volume index ≥35 ml/m^2^, n (%) | 98 (64.5) | 38 (79.2) | 0.057 |
| AAD (class I or III), n (%) | 117 (77.0) | 46 (95.8) | **0.003** |
| Beta-blocker, n (%) | 12 (7.9) | 9 (18.8) | **0.032** |
| ACE inhibitor or ARB, n (%) | 34 (22.4) | 11 (22.9) | 0.937 |
| Cryoballoon applications | 8.6 ± 4.5 | 9.7 ± 5.9 | 0.182 |
| Cryoballoon application time, min | 20.0 ± 7.4 | 21.8 ± 7.8 | 0.154 |
| Nadir balloon temperature, °C |  |  |  |
| LSPV | 50.1 ± 5.3 | 49.6 ± 7.5 | 0.669 |
| LIPV | 44.9 ± 5.2 | 43.3 ± 5.5 | 0.083 |
| RSPV | 51.6 ± 5.7 | 50.7 ± 7.6 | 0.414 |
| RIPV | 46.8 ± 7.5 | 44.5 ± 8.0 | 0.079 |
| Procedure time, min | 81.0 ± 20.8 | 88.0 ± 19.7 | **0.048** |
| LA dwelling time, min | 52.3 ± 16.8 | 56.4 ± 16.1 | 0.160 |
| Fluoroscopy time, min | 25.8 ± 10.7 | 29.5 ± 9.1 | **0.038** |

Values are expressed as n (%), mean ± SD, or median with interquartile range.
BMI = body mass index; TIA = transient ischemic attack; AF = atrial fibrillation; LVEF = left ventricular ejection fraction; LA = left atrium; ACE = angiotensin-converting enzyme; ARB = angiotensin-receptor blocker; CHA_2_DS_2_VASc score = congestive heart failure, hypertension, age ≥75 years, diabetes mellitus, prior stroke or transient ischemic attack or thromboembolism, vascular disease, age 65–74 years, sex category.
