# Supplementary figures and images for: Efficacy and safety of cryoballoon pulmonary vein isolation for paroxysmal and persistent atrial fibrillation: A comparison with radiofrequency ablation
Source: PLoS One. 2022 Jul 27;17(7):e0265482. doi: 10.1371/journal.pone.0265482 (PMC9328506; doi:10.1371/journal.pone.0265482)

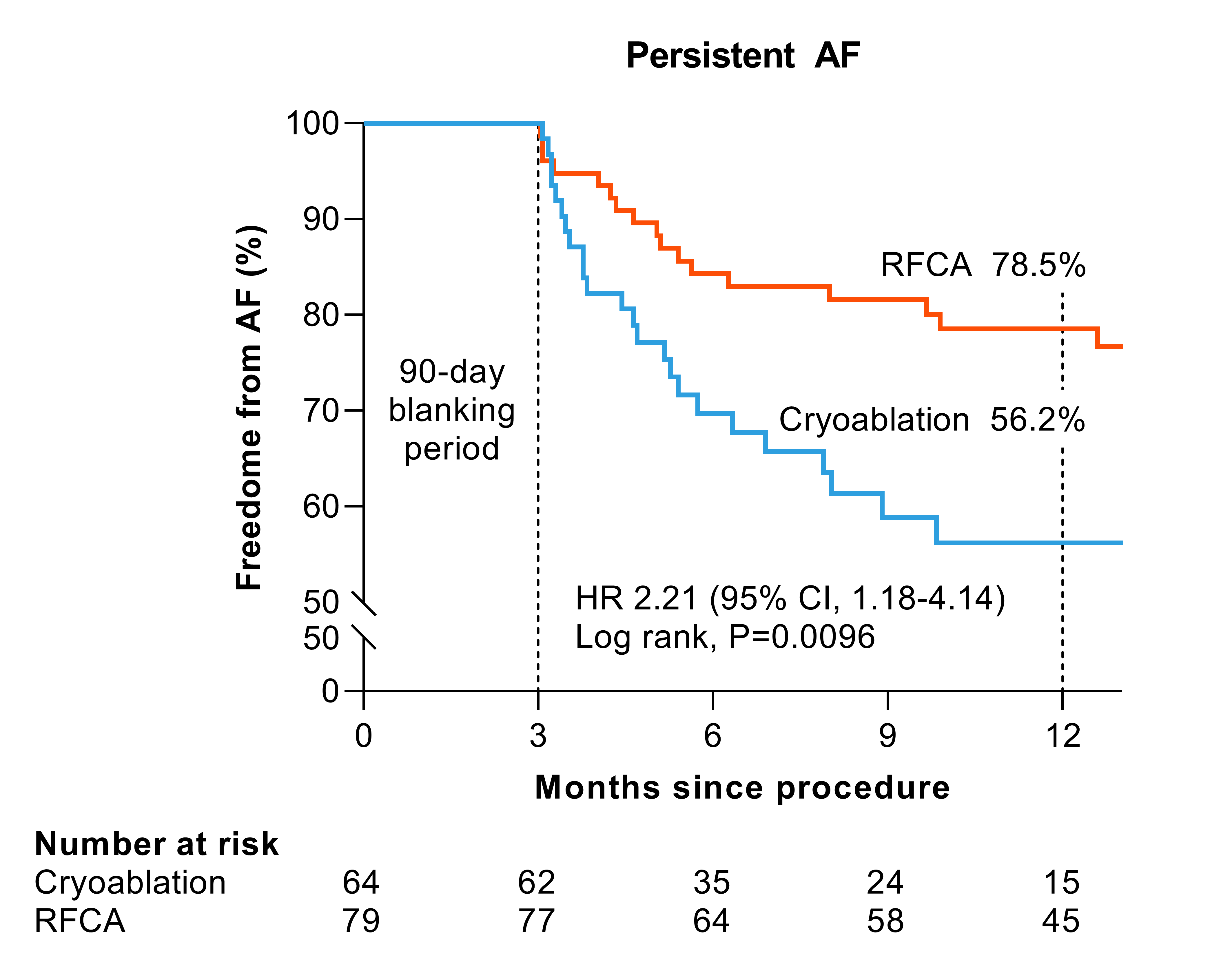

Supplement: S1 Fig — (TIF) [file pone.0265482.s003.tif]
